# Mechanical thrombectomy for inferior vena cava tumor thrombus secondary to renal cell carcinoma

**DOI:** 10.1016/j.jvscit.2024.101626

**Published:** 2024-09-11

**Authors:** Calyb Austin, Rohan McLachlan, Laurencia Villalba

**Affiliations:** a Faculty of Medicine, University of Wollongong, Wollongong, Australia; b Department of Vascular Surgery, Wollongong Hospital, Wollongong, Australia

**Keywords:** Renal cell carcinoma, Thrombectomy, IVC involvement

## Abstract

This case describes the use of percutaneous mechanical thrombectomy to remove caval tumor thrombus in a 55-year-old male with renal cell carcinoma, using the Inari CloTriever system. The procedure was fast, safe, and effective, with complete tumor thrombus removal and no complications. This case demonstrates the expanding indications for large-bore thrombectomy devices offering minimally invasive options in place of high-risk open interventions.

Renal cell carcinoma (RCC) is the most common form of renal cancer. It is the sixth and tenth most frequent oncological diagnosis in women and men, respectively.^1^ A hallmark of this disease is the predisposition for tumor thrombus to spread intraluminally to the renal vein and inferior vena cava (IVC).^2^

Single-session percutaneous mechanical thrombectomy is becoming first-line management for femoro-ilio-caval deep venous thrombosis (DVT).^3^ This is because it is superior to open thrombectomy and catheter directed lysis in terms of safety, efficacy, and overall cost.^4^^,^^5^ Single-session non-lytic thrombectomy has proven successful in the management of iliofemoral thrombosis by complete removal of thrombus. This provides immediate symptom relief, reduces length of hospital stay, and minimizes long-term sequale.^6^ It also avoids serious complications such as major bleeding.^7^ The CloTriever system (Inari Medical) has been shown to be safe and effective for the management of iliofemoral DVTs with the adjunct of the Protrieve sheath (Inari Medical), which provides embolic protection.^6^, ^7^, ^8^, ^9^, ^10^, ^11^ Percutaneous large bore thrombectomy for tumor thrombus extending into the IVC has been previously reported with successful outcomes.^8^^,^^11^, ^12^, ^13^, ^14^, ^15^, ^16^ To the authors’ knowledge, there has been one additional case of the Inari system used for IVC tumor thrombus secondary to RCC with positive outcomes; however, the Protrieve sheath (Inari Medical) was not utilized in this instance.^11^

We present a case, with the patient’s consent, of percutaneous thrombectomy of renal tumor thrombus extending into the IVC and report on operative time, blood loss, length of stay, and complications.

## Case report

A 55-year-old male presented with painless, recurrent frank hematuria. He denied constitutional symptoms, leg pain, or edema. His background included hypertension, chronic obstructive pulmonary disease with an oxygen requirement, and intravenous drug use in the distant past. His medications included candesartan 8 mg daily, amlodipine 10 mg daily, tadalafil 20 mg daily, a combined corticosteroid inhaler, and he was on a methadone program.

**Fig 1:**
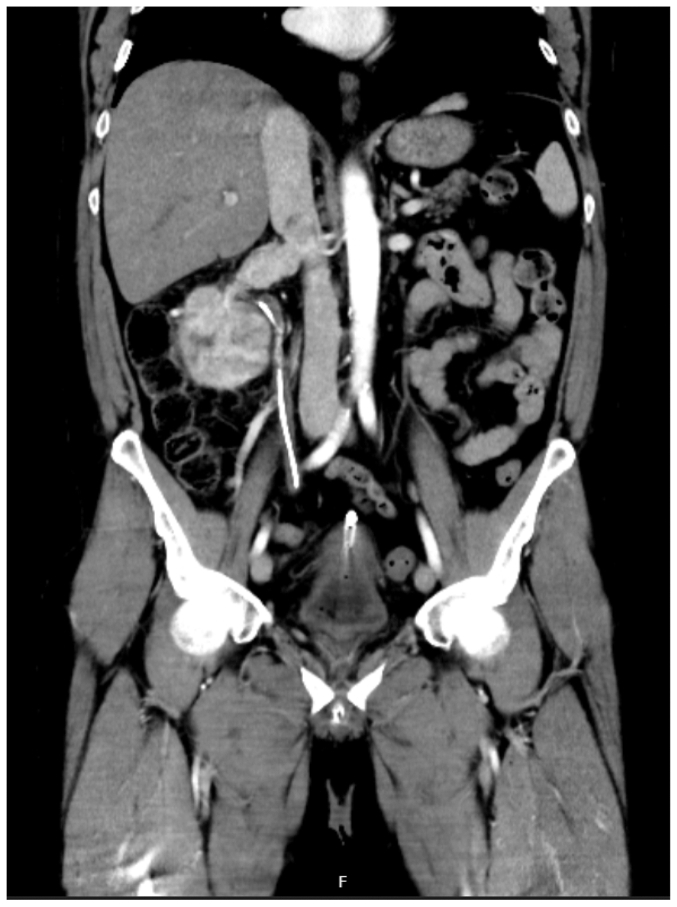
A computed tomography (*CT*) intravenous pyelogram demonstrating a distended and occluded right renal vein, and filling defects evident with the pararenal inferior vena cava (*IVC*) extending to the level of the caudate lobe.

**Fig 2:**
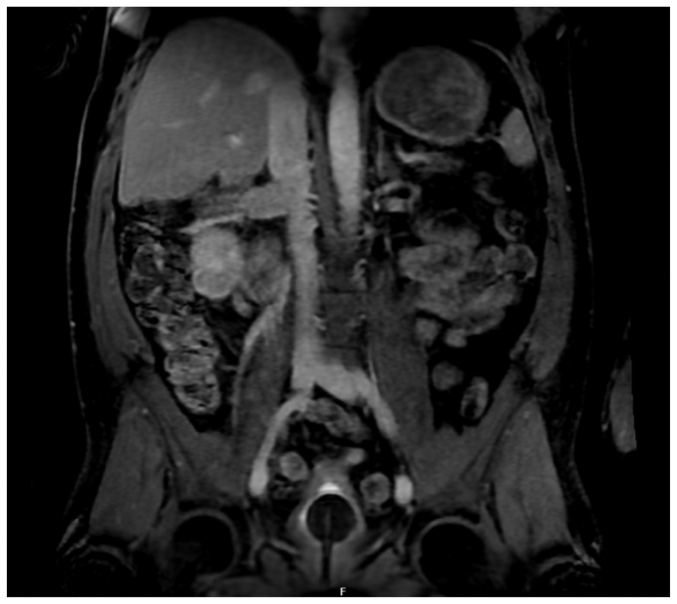
A magnetic resonance angiogram (*MRA*) demonstrating filling defects within the right renal vein, which was distended and occluded, and filling defects in the pararenal inferior vena cava (*IVC*).

His glomerular filtration rate was 76 mL/min/1.73m^2^, and hemoglobin was 93 g/L. A computed tomography (CT) intravenous pyelogram revealed a locally invasive mass in the right kidney spreading to the right renal vein and a filling defect in the pararenal IVC (Fig 1). A magnetic resonance angiogram (MRA) demonstrated the lesion in the inferior pole of the kidney and a right renal vein that was occluded and distended (Fig 2). The lesion extended into the IVC to the level of the caudate lobe consistent with level 2 tumor thrombus.^17^ There did not appear to be propagation of thrombus distally or proximally. A CT of the chest, abdomen, and pelvis did not demonstrate metastatic disease. He was not investigated specifically for pulmonary embolism.

The outcome of a multidisciplinary meeting between the urology, vascular surgery, and oncology teams was to proceed with a combined open right radical nephrectomy and percutaneous IVC thrombectomy with the objective of minimizing vascular risks including embolization, bleeding, and the risks associated with IVC clamping including hemodynamic instability and thrombosis.

**Fig 3:**
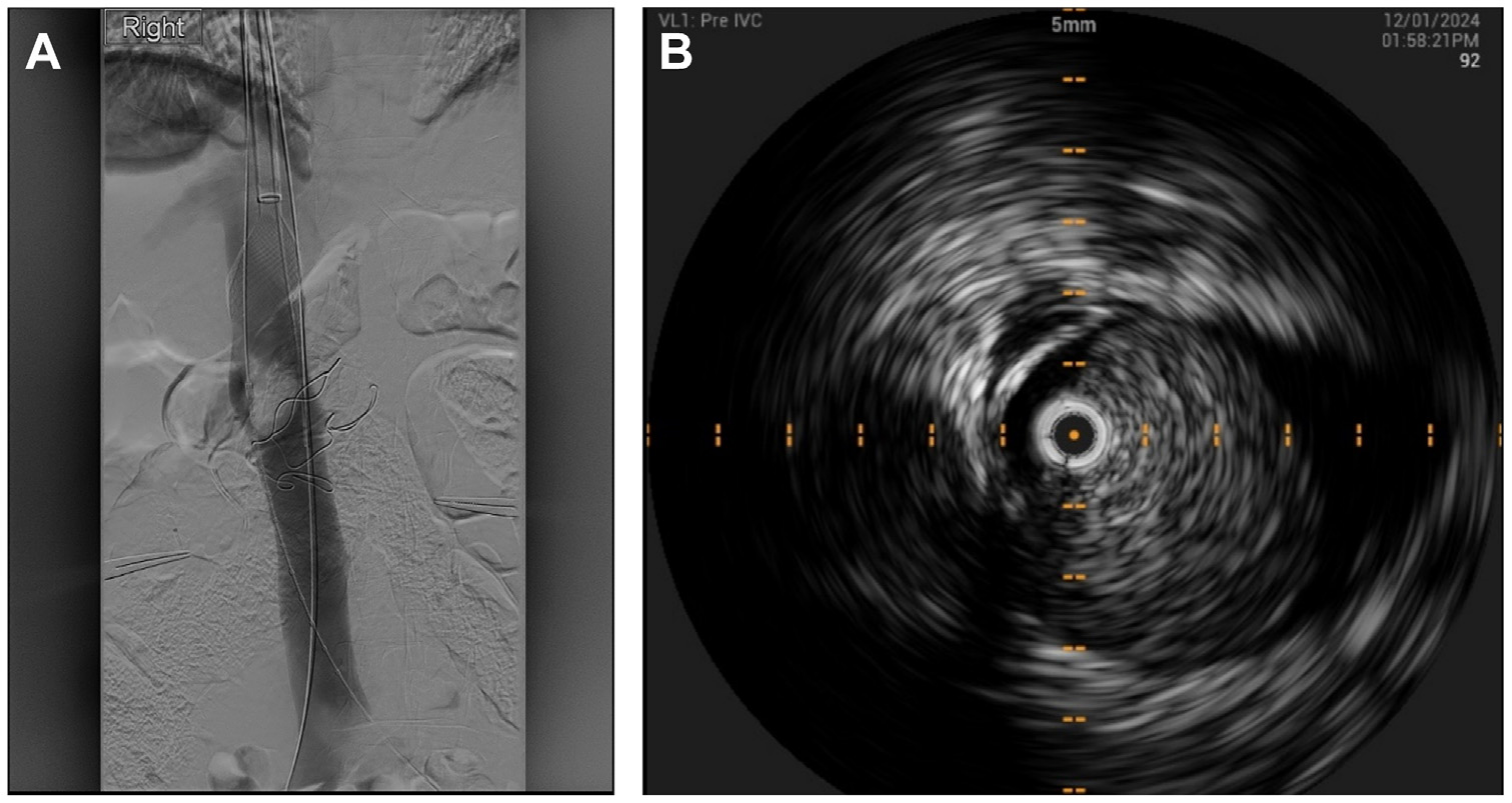
**A,** Digital substraction venography demonstrating a large immobile intraluminal mass in the pararenal inferior vena cava (*IVC*). There is no evidence of a distal or proximal filling defect, and it does not appear to invade the IVC wall. **B,** Intravenous ultrasound (*IVUS*) of the pararenal IVC demonstrating a markedly narrowed caval lumen with a large luminal mass causing near occlusion.

**Fig 4:**
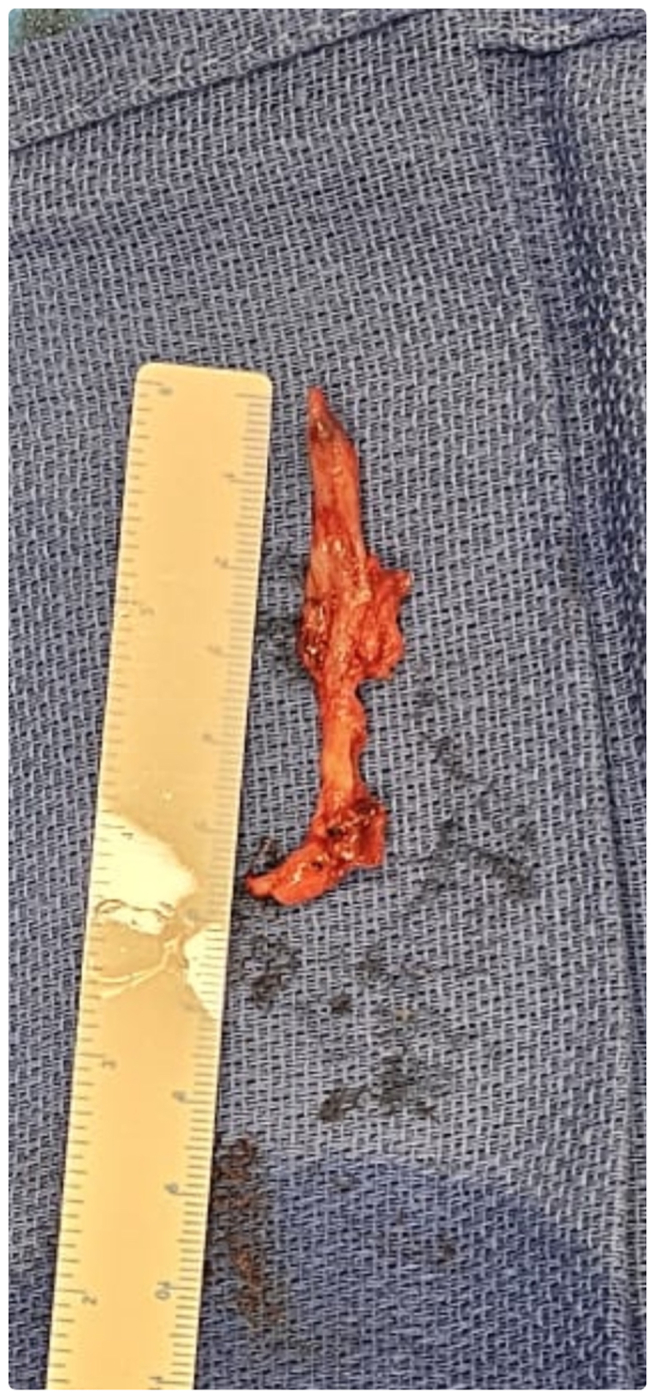
Photograph of extricated inferior vena cava (*IVC*) tumor thrombus measuring 1 × 5 cm.

**Fig 5:**
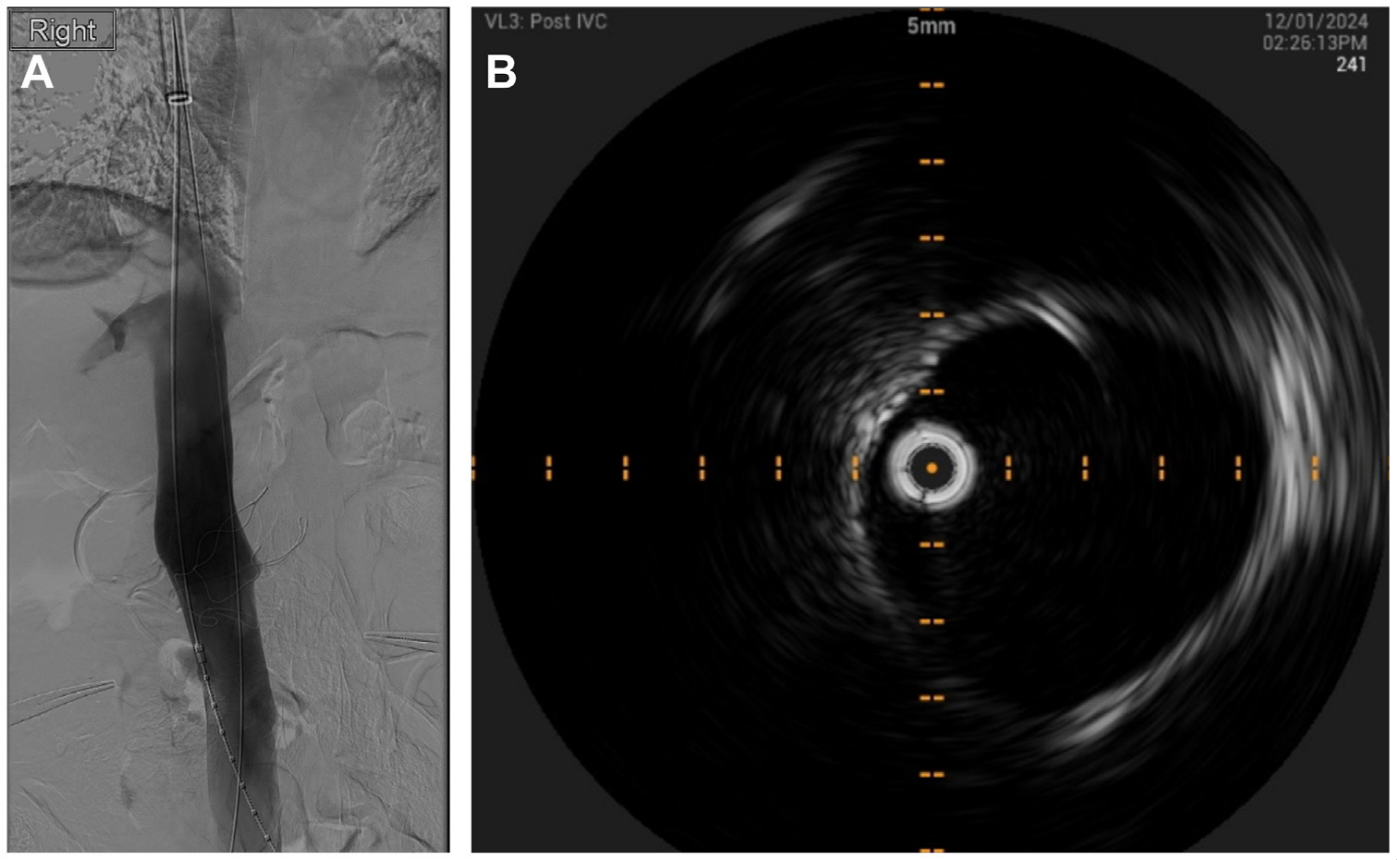
**A,** Digital subtraction venography demonstrating complete clearance of the pararenal inferior vena cava (*IVC*) mass following endovascular mechanical thrombectomy. **B,** Intravenous ultrasound (*IVUS*) of the pararenal inferior vena cava (*IVC*) demonstrating a widely patent caval lumen following endovascular mechanical thrombectomy.

The urology team mobilized the right kidney via a midline laparotomy and ligated the ureter and two right renal arteries. A vessel loop was positioned around the right renal vein, which was evidently occluded. The vascular surgeons gained ultrasound guided access of the right internal jugular vein (IJV) and bilateral common femoral veins (CFV) and deployed pre-closure using Prostyle (Abbott Vascular Devices) closure devices. Fourteen cm × 10 Fr sheaths (Cook Medical) were placed in each access, followed by intravenous heparin loading with 5000 IU. A 24 Fr Protrieve sheath (Inari Medical) was inserted through the right IJV, and the funnel was positioned in the proximal IVC, 3 cm from the right atrium. A 16 Fr CloTriever XL sheath (Inari Medical) was inserted via the right CFV access over an Amplatz (Boston Scientific) wire that was used as a through and through wire via the Protrieve sheath (Inari Medical), with the funnel deployed at the level of the right external iliac vein. The left CFV access was used for the 0.35 intravascular ultrasound (IVUS) (Philips Volcano). Digital subtraction venography and IVUS demonstrated a large immobile intraluminal mass in the pararenal IVC. It was moderately hyperechoic, with no evidence of distal or proximal thrombosis. The mass did not appear to invade the wall of the IVC (Fig 3, *A* and *B*). The CloTriever XL catheter (Inari Medical) was introduced and positioned with the coring element proximal to the IVC mass. A single pass of CloTriever catheter (Inari Medical) was performed from the proximal IVC and out of the right CFV sheath. A 1 cm × 6 cm soft tissue mass was retrieved, compressed from being extracted via the sheath, with no associated thrombus (Fig 4). The CT sheath and the PT sheath were aspirated. Thrombotic material was suctioned from the CT sheath but not from the PT sheath. Post intervention IVUS and venogram demonstrated complete removal of the mass and a widely patent IVC with no filling defects (Fig 5, *A* and *B*). The right renal vein was then clamped at the renocaval confluence, which had become patent. The vein was transected, and a small volume of tumor thrombus was retrieved before ligation of the stump with 4/o prolene. The nephrectomy was completed, and the laparotomy closed. The sheaths were removed, and the Proglide sutures were used to close all access sites. The vascular component of the procedure was 45 minutes, and the estimated blood loss was 200 mL from the sheath aspiration.

Postoperatively, the patient was placed on enoxaparin (1 mg/kg twice daily dosing) for two weeks as a prophylactic measure to prevent DVT secondary to intraoperative venous manipulation. A CT pulmonary angiogram on day two did not demonstrate any pulmonary embolism. This was completed to rule out pulmonary embolism as this was the first time the procedure was performed in this center without IVC clamping. The patient was discharged on day six with no evidence of complication.

Duplex ultrasound and MRA at 2 weeks showed no residual thrombus within the IVC. Histological specimens from the renal mass and IVC mass were consistent with clear cell renal carcinoma, staging pT3b.

## Discussion

A hallmark of RCC is the predisposition for tumor thrombus invasion into the IVC occurring in 4% to 10% of cases.^18^^,^^19^ Patients who have RCC with vascular invasion have a poor prognosis due to the associated risks such as embolization and thrombus propagation with a median survival of 5 months.^19^^,^^20^ For this reason, urgent evaluation and surgical management are paramount.^1^ The surgical objectives are complete resection of the tumor and associated caval thrombi while preventing embolization.^19^

The standard approach is open nephrectomy and open caval thrombectomy with or without caval reconstruction.^21^^,^^22^ The vascular component of the procedure carries substantial potential risks including major blood loss, embolization of tumor thrombus, air embolus, severe hemodynamic instability due to the need for caval cross-clamping, risk of caval thrombosis, and acute kidney injury also due to cross-clamping.^15^^,^^23^

A percutaneous approach with adjunctive embolic protection has the potential to minimize those risks by avoiding the need for caval clamping. This is because extensive dissection, venotomy, and closure are not required, which minimizes the potential blood loss as well as the risk of thrombosis and acute kidney injury, while also being a faster procedure.^15^^,^^22^ The CloTriever system is a mechanical thrombectomy device designed for the removal of DVT with large clot burden.^24^ It is a sheathed catheter that uses a nitinol mesh funnel to remove clot and a nitinol coring element and braided collection bag, which extracts the clot within the sheath.^25^ It has demonstrated success in management of iliocaval venous thrombi with complete or near complete removal of thrombus in 91.2% of patients. Nearly all procedures are performed in a single session (median procedural time, 59-75 minutes) with negligible blood loss (mean of 50 mL).^26^

The Protrieve Sheath funnel provides atraumatic wall apposition for complete embolic capturing and provides rapid removal of any captured embolic material due to the large-bore manual aspiration mechanism. It provides a large surface area, which is suitable for caval thrombi. In an open approach, the average blood loss is 2500 ± 2827 mL, the median operative duration is 5.5 hours, and the average length of stay is 9 to 12.5 days.^27^^,^^28^ In comparison to this case, the estimated total blood loss was 600 mL, the total operative time was 4.5 hours, the vascular surgery component took 45 minutes, and the patient was hospitalized for 6 days. The estimated blood loss during the vascular portion was mostly attributed to sheath aspiration to monitor for thrombus. In non-tumor thrombotic cases, the blood could have been returned using the FlowSaver Blood Return System (Inari Medical); however, it was avoided in this case to minimize potential seeding risk.

The successful outcome of this case using the Inari system is in line with other case reports of caval thrombi secondary to RCC, cervical cancer, sacral chordoma, hepatocellualar carcinoma, pancreatic cancer, liposarcoma, seminoma, and adenocarcinoma. The speed, safety, and efficacy of the procedure continues to be reported consistently in the literature.^8^^,^^11^, ^12^, ^13^, ^14^, ^15^, ^16^ This case had the added benefit of utilizing the Protrieve Sheath for embolic protection, which successfully prevented pulmonary embolization.

The use of mechanical thrombectomy has limitations and risks. The Protrieve sheath (Inari Medical) can be deployed 3 cm distal to the right atrium to allow for deployment of the funnel. Therefore, grade IV tumors that are proximal within 3 cm of the right atria or include atrial thrombus would not allow for deployment of the Protrieve sheath.^18^

The risk of microscopic seeding risk is not well-defined in traditional surgical techniques, and the authors acknowledge the theoretical risk of microscopic seeding using the mechanical thrombectomy system as the tumor traverses the sheath. Patients with RCC have a high rate of metastastic disease, with 30% to 50% of patients developing pulmonary metastases, and one-third will develop metastatic disease after traditional radical nephrectomy techniques for localized RCC.^29^^,^^30^ Venous thrombus consistency (solid vs friable) has been suggested to impact on prognosis in retrospective studies.^31^^,^^32^ Future studies should aim to address the efficacy and safety of the Inari system (Inari Medical) in the oncology setting, considering the thrombus consistency. Patients with intermediate- to high-risk disease (pT2G4, pT3 or higher, nodal involvement, or resected M1 disease) should be offered adjuvant immune checkpoint inhibitors.^33^^,^^34^ The use of adjuvant immunotherapy may attenuate the risk of recurrence from theoretical microscopic seeding.

In the authors’ opinion, percutaneous large bore thrombectomy with adjunctive embolic protection should be a considered intervention for caval tumour thrombi.

## Conclusion

Percutaneous thrombectomy using the Inari CloTriever system for managing renal tumor thrombosis extending into the IVC is fast, safe, and effective. Avoiding caval dissection, cross-clamping, venectomy, and IVC repair minimizes vascular risk, blood loss, and operative times. Further studies on percutaneous thrombectomy for IVC tumor thrombus will be valuable in validating its utility and expanding its application in similar cases.

## Disclosures

None.
